# Investigation of Variants in UCP2 in Chinese Type 2 Diabetes and Diabetic Retinopathy

**DOI:** 10.1371/journal.pone.0112670

**Published:** 2014-11-14

**Authors:** Yinchen Shen, Zujia Wen, Ning Wang, Zhi Zheng, Kun Liu, Xin Xia, Qing Gu, Yongyong Shi, Xun Xu

**Affiliations:** 1 Department of Ophthalmology, Shanghai First People's Hospital, Shanghai Jiao Tong University, Shanghai, People's Republic of China, Shanghai Key Laboratory of Fundus Disease, Shanghai, People's Republic of China; 2 Bio-X Institutes, Key Laboratory for the Genetics of Developmental and Neuropsychiatric Disorders (Ministry of Education), Shanghai Jiao Tong University, Shanghai, People's Republic of China; The University of Hong Kong, Hong Kong

## Abstract

**Purpose:**

The aim of this study was to investigate variants in *UCP2* genes in type 2 diabetes mellitus (DM) and diabetic retinopathy (DR) in Chinese population.

**Materials and Methods:**

We conducted a single nucleotide polymorphism-based and haplotype-based case-control study between the variants of *UCP2* and DM and between the variants of *UCP2* and DR in 479 Chinese patients with type 2 DM and 479 control subjects without DM. Two SNPs (rs660339 and rs659366) were selected as genetic markers.

**Results:**

The risk allele C at *UCP2* rs660339 was closely associated with DM in Chinese population. There was significant difference in rs660339 between DM and controls (P = 0.0016; OR [95%CI]  = 1.37 (1.14–1.65)). Subjects who were homozygous of the C allele were more likely to develop DM. The frequency of C allele was higher in DM (58%) than in control (51%). But this locus didn't have a definite effect on the onset of non-proliferative diabetic retinopathy (NPDR) (P = 0.44; OR [95%CI]  = 0.80 (0.56–1.14)) and proliferative diabetic retinopathy (PDR) (P = 1.00; OR [95%CI]  = 0.99 (0.74–1.34)) comparing to subjects with DM without retinopathy (DWR), respectively. Moreover, the *UCP2* rs659366 polymorphism showed no significant difference between DM and control (P = 0.66; OR [95%CI]  = 1.10 (0.91–1.32)). However, there was a significant difference between PDR and DWR (P = 0.016; OR [95%CI]  = 0.66 (0.49–0.90)), but there was no difference between NPDR and DWR (P = 1.00; OR [95%CI]  = 0.96 (0.67–1.37)). Participants who carried the G allele at rs659366 were more likely to develop PDR. For the haplotype, C-A was present more frequently in DM than in control (16% vs 7%), indicating that it was risky, and T-A was present less in DM than in control (29% vs 35%). Haplotype frequencies in DR and DWR showed no significant difference (P = 0.068).

**Conclusion:**

It was indicated that *UCP2* may be implicated in the pathogenesis of type 2 DM and DR in Chinese population.

## Introduction

Diabetic retinopathy (DR) is a common sight-threatening microvascular complication affecting patients with diabetes mellitus (DM) and it is a major cause of new cases of blindness [1]. In the United States, an estimated 25.8 million children and adults or approximately 8.3% of the population, have diabetes [2]. Meanwhile, according to a recent epidemiological survey in China, the overall prevalence of diabetes was estimated to be 11.6% in Chinese adult population [3]. With such incidence, and prevalence of DM increasing worldwide, DR is expected to reach epidemic proportions.

Many diabetes-induced metabolic abnormalities are implicated in the development of DR, and appear to be mostly influenced by elevated oxidative stress [4]. Consequently, different candidate genes of oxidative stress were paid special attention to [5]. Several genetic and functional studies have implicated the important role of oxidative stress genes in the pathogenesis of retinopathy in diabetes [6]–[8]. However, the exact mechanism underlying its development remains unclear. There is considerable evidence that in patients with diabetes, mitochondrial reactive oxygen species (ROS) generation, especially at high blood glucose levels, is one of the main sources of oxidative stress [9], [10]. Some antioxidant defense mechanisms exist to neutralize ROS and prevent harm. One of these is represented by human uncoupling proteins (UCPs).

UCPs are mitochondrial transporters present in the inner membrane of mitochondria which are considered pivotal regulators of energy and glucose homeostasis [11]. They belong to the mitochondrial anion carrier gene family and as suggested by their name, UCPs can uncouple ATP production from mitochondrial respiration by causing proton leak [12]. Recent studies demonstrated that UCPs can prevent mitochondrial hyperpolarization and formation of ROS [13]. A group of five different UCPs have been identified. These proteins have distinct tissue distributions and functions. Among them, *UCP2* is widely expressed in almost all mammalian tissues including white adipose tissue, liver, kidney, pancreatic islets, macrophages and retinal endothelial cells and pericytes [14]. Recently, the relationships between *UCP2* polymorphisms and susceptibility for type 2 DM [15]–[17], obesity [18] and cardiovascular disease [19] have been investigated. Several variants including Ala55Val polymorphism (rs660339) in the exon 4 and −866G/A polymorphism (rs659366) in the promoter region were paid specific attention to, however, the results were contradicting [15]–[17]. Limited studies about the association between *UCP2* polymorphisms and DM in Chinese population have found no significant differences [20]. In addition, no study has investigated the relationship between these two polymorphisms and the risk of DR in Chinese population.

Considering the role of *UCP2* in the protection against oxidative stress, we conducted an association analysis between variants of *UCP2* and DM and between variants of *UCP2* and DR in subjects with type 2 DM in Chinese population.

## Materials and Methods

### Subjects

The study population comprised of 479 unrelated patients with type 2 diabetes mellitus (mean age ± SD, 58.80±12.02; ratio of male to female, 45.3∶54.7) and 479 unrelated controls subjects without DM (mean age ± SD, 58.74±12.22; ratio of male to female, 42.4∶57.6), all of Han Chinese ethnicity. The subjects with DM were randomly recruited from the data bank in the department of ophthalmology of Shanghai First People's Hospital, Shanghai Jiao Tong University. The diagnosis of diabetes was based on criteria outlined by the American Diabetes Association in 1997. Control subjects without DM were chosen from a population of the outpatient of Shanghai First People's Hospital, Shanghai Jiao Tong University. The inclusion criteria of the control group was healthy blood donors of Chinese ancestry who did not have DM or family history for this disease. This study was approved by the Ethics committee of Shanghai First People's Hospital, Shanghai Jiao Tong University, Shanghai, China (permit number: 2013KY095). An informed consent process was established following the guidelines of the Helsinki Declaration, and consent forms were signed by all subjects.

We obtained blood samples from all the 958 subjects for DNA extraction. All subjects with DM underwent a standard ophthalmic examination, including measurement of visual acuity, slit-lamp biomicroscopy and dilated fundus examination. The evaluation of DR was according to the diagnostic criteria of the American Academy of Ophthalmology (AAO) 2001 Annual Meeting.

Among the 479 subjects with DM, there were 324 patients with DR (n = 215 of proliferative diabetic retinopathy (PDR) and n = 109 of non-proliferative diabetic retinopathy (NPDR)), 155 subjects with DM without retinopathy (DWR). The data of patients' age, gender, duration of diabetes, fasting blood glucose levels, systolic blood pressure, diastolic blood pressure, height and weight were all recorded.

Age, gender, systolic blood pressure, diastolic blood pressure and BMI were comparable in the control, DWR and DR groups. Besides, there was no significant difference in the duration of diabetes and the fasting blood glucose between DWR and DR groups (P>0.05). Demographic characteristics of all patients included in the study were shown in Table 1.

**Table 1:** Demographic characteristics of the patients with DM and control subjects.

	Control	DWR	DR
No. of subjects	479	155	324
Male: female	203: 276	78: 77	139: 185
Age (years)	58.74±12.22	60.15±10.90	58.15±12.52
Duration of diabetes (years)	—	10.61±6.77	11.42±6.93
Fasting blood glucose (mmol/L)	—	8.40±2.52	8.76±3.11
Systolic blood pressure (mmHg)	128.55±16.66	129.72±16.19	130.63±16.52
Diastolic blood pressure (mmHg)	76.90±9.24	78.50±8.30	77.89±9.29
BMI (kg/m^2^)	24.33±3.56	24.69±3.19	24.90±3.03

The results are expressed as mean ± SD.

### Genotyping

We selected rs660339 in the exon 4, and rs659366 in the promoter region of *UCP2* gene for our study. Genomic DNA was extracted from venous blood using standard phenol-chloroform extraction for the genotyping of these two polymorphisms. PCR amplifications producing two genomic segments, rs659366 and for rs660339, were performed on the GeneAmp PCR 9700 System (Applied Biosystems, ABI, USA) in a 15.0 µl reaction containing 10 ng genomic DNA,1.2 U Taq polymerase, 0.2 µl of each primer (10 PM), 1.5 µl PCR buffer (10×, Qiagen), and 1.5 µl dNTPs (each 2 mM). Amplification conditions consisted of an initial 3 min at 95°C, 35 cycles of 30 sec at 94°C, and 30 sec at 57°C for the two segments. We then genotyped the two variants using DNA sequencing on an ABI 3100 genetic analyzer using the BigDye Terminator Cycle Sequencing Kit (Applied Biosystems, ABI, USA). The primers and the sequence number definition of *UCP2* were constructed on the basis of NC_000006. Primer sequences were listed in the Table S1.

### Statistical analysis

The parameters, allele and genotype frequencies, Hardy-Weinberg equilibrium (HWE), pair-wised linkage disequilibrium, and haplotype analysis were conducted online using a software platform called SHEsis (http://analysis.bio-x.cn) [21]. A case-control format was adopted for data analysis. The P values of allele and genotype frequencies were obtained based on chi-square distribution. We adjusted the P values using the Bonferroni correction. The threshold value of HWE was set at 0.05. Haplotype analysis was conducted using the PLEM algorithm, and those haplotypes that exhibited a frequency less than 0.03 were abandoned.

## Results

Totally, 479 subjects with DM and 479 controls were enrolled in our study. All of the polymorphisms investigated were proved to be in HWE. The results of HWE testing were showed in the Table S2. The total number of samples genotyped differed between the variants because not all were capable of being amplified with PCR.

Two polymorphisms of *UCP2* (rs660339 and rs659366) were genotyped by sequencing in the 958 subjects. The frequency distribution of the alleles and genotypes were present in Table 2, Table 3 and Table 4.

**Table 2:** Distributions of genotypes and alleles for the two variants in the DM and controls.

SNP site	Samples	N	Allele		χ2	p	OR (95%CI)	Genotype			χ2	P
			**C**	**T**				**CC**	**CT**	**TT**		
**rs660339**	DM	472	551(58%)	393(42%)	11.20	**0.0016**	1.37(1.14–1.65)	166(35%)	219(46%)	87(19%)	10.69	**0.01**
	Control	441	446(51%)	436(49%)				121(28%)	204(46%)	116(26%)		
			**A**	**G**				**AA**	**AG**	**GG**		
**rs659366**	DM	454	411(45%)	497(55%)	0.96	0.66	1.10(0.91–1.32)	97(21%)	217(48%)	140(31%)	1.14	1.00
	Control	448	385(43%)	511(57%)				90(20%)	205(46%)	153(34%)		

P value were calculated between DM and control.

**Table 3:** Distributions of genotypes and alleles for the two variants in the DR and DWR.

SNP site	Samples	N	Allele		χ2	p	OR (95%CI)	Genotype			χ2	P
			**C**	**T**				**CC**	**CT**	**TT**		
**rs660339**	DR	317	366(58%)	268(42%)	0.33	0.57	0.92(0.70–1.22)	106(33%)	154(49%)	57(18%)	1.92	0.38
	DWR	155	185(60%)	125(40%)				60(39%)	65(42%)	30(19%)		
			**A**	**G**				**AA**	**AG**	**GG**		
**rs659366**	DR	305	262(43%)	348(57%)	4.02	**0.045**	0.75(0.57–0.99)	59(19%)	144(47%)	102(34%)	3.89	0.14
	DWR	149	149(50%)	149(50%)				38(26%)	73(49%)	38(25%)		

P value were calculated between DR and DWR.

**Table 4:** Distributions of genotypes and alleles for the two variants in NPDR, PDR and DWR.

SNP site	Samples	N	Allele		χ2	p	OR (95%CI)	Genotype			χ2	P
			**C**	**T**				**CC**	**CT**	**TT**		
rs660339	NPDR	106	115(54%)	97(46%)	1.52	0.44	0.80(0.56–1.14)	33(31%)	49(46%)	24(23%)	1.61	0.90
	PDR	211	251(60%)	171(40%)	0.003	1.00	0.99(0.74–1.34)	73(34%)	105(50%)	33(16%)	2.31	0.62
	DWR	155	185(60%)	125(40%)				60(39%)	65(42%)	30(19%)		
			**A**	**G**				**AA**	**AG**	**GG**		
rs659366	NPDR	102	100(49%)	104(51%)	0.05	1.00	0.96(0.67–1.37)	23(23%)	54(53%)	25(24%)	0.43	1.00
	PDR	203	162(40%)	244(60%)	7.11	**0.016**	0.66(0.49–0.90)	36(18%)	90(44%)	77(38%)	6.93	0.06
	DWR	149	149(50%)	149(50%)				38(26%)	73(49%)	38(25%)		

P value were calculated between NPDR or PDR and DWR, respectively.

When Bonferroni corrections were strictly adopted, there was significant difference in rs660339 between DM and controls (P = 0.0016; OR [95%CI]  = 1.37 (1.14–1.65)). The frequency of C allele was higher in DM (58%) than in control (51%). The percentage of genotype CC at rs660339 were higher in DM (35%) and than in control subjects (28%) (P<0.05). Multiple comparison for the 3 genotypes (CC, CT, TT) showed that there were significant differences between CC and TT (χ2 = 10.688, P = 0.001) and between CT and TT (χ2 = 4.364, P = 0.037), but no difference was found between CC and CT (χ2 = 2.535, P = 0.111). The results of multiple comparison were showed in the Table S3. It suggested that the risk allele C was closely associated with type 2 DM in Chinese population. Moreover, rs659366 showed no significant difference between DM and control (P = 0.66; OR [95%CI]  = 1.10 (0.91–1.32)). We observed no significant difference in genotype distribution at rs659366 between DM and control group (P>0.05) (Table 2).

To investigate the association between *UCP2* variants and DR, we found no significant difference between DR and DWR (P = 0.57; OR [95%CI]  = 0.92 (0.70–1.22)) for rs660339. Nevertheless, for rs659366, there was a significant difference between DR and DWR (P = 0.045; OR [95%CI]  = 0.75 (0.57–0.99)). For the genotype distribution analysis, we found no significant difference between DR and DWR at rs660339 and rs659366 (Table 3).

Then, we further compared the distribution of alleles between DR subtypes (NPDR, PDR) and DWR. For rs660339, we found no significant difference neither between NPDR and DWR (P = 0.44; OR [95%CI]  = 0.80 (0.56–1.14)) nor between PDR and DWR (P = 1.00; OR [95%CI]  = 0.99 (0.74–1.34)). Interestingly, for rs659366, there was a significant difference between PDR and DWR (P = 0.016; OR [95%CI]  = 0.66 (0.49–0.90)). But, there was no difference between NPDR and DWR (P = 1.00; OR [95%CI]  = 0.96 (0.67–1.37)). For the genotype distribution analysis, we found no significant difference among NPDR, PDR and DWR groups at rs660339 and rs659366. However, participants who were homozygous for the G allele at rs659366 were more likely to develop PDR but there was no statistical significance (P = 0.06) (Table 4).

Within *UCP2*, pair-wised linkage disequilibrium analysis in DM case showed rs660339 was in moderate linkage disequilibrium (D′ = 0.52) with rs659366 (Table S4).

For the haplotype analysis, one significant association was noted for C-A haplotype that was present two times frequently in DM than in control (16% vs 7%), indicating that it was risky. Another significant association was T-A haplotype which was present less frequently in DM than in control (29% vs 35%) (Table 5). Moreover, haplotype frequencies in DR and DWR showed no significant difference (P = 0.068) (Table 6).

**Table 5:** The estimated haplotype frequencies in DM and control.

Haplotype	DM	Control	Chi-square	OR(95%CI)	Global P value
C-A	140.5(16%)	58.0(7%)	31.97	2.48(1.79–3.42)	**P<0.001** (*df* = 3, χ2 = 34.76)
C-G	385.5(43%)	361.0(44%)	0.04	0.98(0.81–1.19)	
T-A	263.5(29%)	294.0(35%)	7.11	0.76(0.62–0.93)	
T-G	106.5(12%)	117.0(14%)	1.88	0.82(0.62–1.09)	

**Table 6:** The estimated haplotype frequencies in DR and DWR.

Haplotype	DR	DWR	Chi-square	OR(95%CI)	Global P value
C-A	86.1 (14%)	53.9 (18%)	2.07	0.76(0.52–1.11)	P = 0.068 (*df* = 3, χ2 = 10.21)
C-G	258.9(43%)	127.1 (43%)	0.04	1.03(0.78–1.36)	
T-A	168.9(29%)	95.1(32%)	1.28	0.84(0.62–1.14)	
T-G	84.1(14%)	21.9(7%)	8.55	2.06(1.26–3.37)	

## Discussion

DM and its chronic complications are complex diseases associated with both genetic and environmental risk factors [22]. Considering the important role of *UCP2* in ROS formation by mitochondria, the relationship between *UCP2* locus and susceptibility for DM and its chronic complications [15]–[17], [23]–[26] has been investigated. Over the past few years, several studies have suggested that there was an association between the *UCP2*-866G/A (rs659366) and Ala55Val (rs660339) polymorphisms and the risk of type 2 DM [15]–. However, the results showed significant between-study variation. Prior studies examining these two variants in the *UCP2* gene have produced controversial results [15]–[17]: population of different races, ethnic backgrounds, and environmental exposures might influence the final result. Moreover, the evidence in Chinese population was quite limited. Recent study conducted in Hubei Han Chinese have reported no significant association between *UCP2* polymorphisms and type 2 DM [20]. Therefore, we found it still necessary to enlarge the sample size and conduct further investigation about the effects of *UCP2* genetic polymorphisms on type 2 DM.

In this study, we genotyped rs659366 and rs660339 of *UCP2* in 958 participants. The SNP analysis demonstrated that the C allele of rs660339 had significantly higher frequency in DM (P = 0.0016; OR [95%CI]  = 1.37 (1.14–1.65)) than in control subjects, indicating that the risk C allele was related with the onset of DM in Chinese population. Meanwhile, participants who were homozygous for the C allele at rs660339 were more likely to develop DM. Haplotype analysis demonstrated that C-A haplotype was present two times more in DM than in control, while T-A haplotype was present less frequently in DM than in control, indicating that C was risky. The result of haplotype analysis was in accordance with those of allele and genotype analysis between DM and control. Our result complied with prior studies conducted by Lee et al. [11] and Vimaleswaran et al. [27] in Asian ethnicity. Nevertheless, the *UCP2* rs659366 polymorphism showed no significant difference between DM and control (P = 0.66; OR [95%CI]  = 1.10 (0.91–1.32)). Other studies conducted in Asian ethnicity have reported different results [11], [27]–[30]. Some indicated that patients with G alleles at rs659366 were more likely to suffer from DM [11], and the others showed no significant difference [27]–[30]. According to a recent meta-analysis of seventeen published articles, rs659366 was not a candidate for susceptibility to type 2 DM in any ethnic population, however, rs660339 was type 2 DM susceptibility loci in population of Asian, but not European descent [31]. Our study has consolidated these findings in Chinese population.

As a severe sight-threatening microvascular complication of DM, DR represents a major cause of new cases of blindness in adults [32]. There already had evidence showed that longer duration of diabetes, poorer control of blood glucose and elevated blood pressure were the major factors responsible for the onset and progression of DR. However, DR still occurs in subjects with normal glucose and blood pressure [33]. In addition, the prevalence of DR also showed racial differences and familial clustering indicating that genetic factors may have a significant effect in promoting the severity and the speed of onset of retinopathy in patients with DM [34]. Thus, identifying the genes contributing to the susceptibility of DR is quite important. Experimental research demonstrated that an overproduction of ROS was a triggering cause in the pathogenesis of diabetic complications. *UCP2* can protect retinal vascular endothelial cells from damage by inhibiting the overproduction of mitochondrial ROS [35], a phenomenon that revealed the important role of *UCP2* in DR. Although the genetic association between *UCP2* and DM has already been investigated, how *UCP2* polymorphsms influence the onset of DR still requires more evidences based on case-control genetic study. Crispim D et al. [23] has reported an association between the polymorphisms of *UCP2* and PDR in subjects with both type 2 and type 1 DM in European ancestry. However, there was no evidence about the relationship between *UCP2* and DR in Asian population. Therefore, we examined the two common *UCP2* gene polymorphisms, rs660339 and rs659366 variants, in Chinese participants to try to confirm this association.

For rs660339, we found no significant difference for the SNP analysis between DR and DWR. Although Crispim D et al. [23] indicated that type 2 DM patients with PDR presented higher frequencies of the T allele than type 2 DM patients without this complication, we observed no significant difference between NPDR or PDR and DWR, respectively. We guess that their result might only be limited to European ethnicity. Besides, for *UCP2* rs659366 polymorphism, there was a significant difference between DR and DWR. It was interesting that when we compared the SNP distributions in DR subtypes and DWR, we observed a significant difference between PDR and DWR, but there was no difference between NPDR and DWR. Participants who were homozygous for the G allele at rs659366 were more likely to develop PDR, indicating that the risk G allele was associated with the onset of PDR. When Bonferroni corrections were strictly adopted, we found that haplotype frequencies in DR and DWR showed no significant difference. In previous studies, Rudofsky et al. [24] reported no association of the rs659366 polymorphism with DR in a sample of Caucasian type 2 DM patients. However, Crispim D et al. [23] found that type 2 DM patients with PDR presented higher frequency of A allele. Our finding contradicted with prior studies in European ethnicity probably due to a racial difference, and our preliminary results need further consolidation by larger sample studies. It is worth pointing out that most studies focused on the genetic epidemiology of *UCP2* gene, and its function or protein expression changes by the influence of gene mutations were rarely investigated. Recently, one study indicated that the presence of the −866A/55Val/Ins haplotype was associated with decreased *UCP2* gene expression in human retina [36], which shed light on further research.

In conclusion, we examined the frequencies of two common polymorphisms in *UCP2*, rs660339 and rs659366, in the type 2 DM patients consist of NPDR, PDR, DWR and also control subjects without DM. The result indicated that homozygous of the C allele at *UCP2* rs660339 were more likely to develop DM, but this locus doesn't have a definite effect on the onset of NPDR and PDR. Moreover, the *UCP2* rs659366 polymorphism demonstrated a modest relationship with DM, but the DR patients who were homozygous for the G allele at rs659366 were more likely to develop PDR. Although the genetic susceptibilities to DR and to DM were sometimes similar, the difference still existed.

SNPs in the *UCP2* may be implicated in the pathogenesis of type 2 DM and DR in Chinese population. As far as our knowledge concerns, this is the first genetic study showing that *UCP2* polymorphisms are associated with DR in Asian population. However, this is a preliminary study and further functional studies are required to confirm its effect in human retinal cells.

## Supporting Information

Table S1 **Primers used in PCR for each of the two polymorphisms.**
(DOCX)

Table S2 **Hardy-Weinberg equilibrium testing.**
(DOCX)

Table S3 **Multiple comparison of genotypes distribution in rs660339 between DM and control.**
(DOCX)

Table S4 **Linkage disequilibrium between SNPs in our samples, showing D'(above the diagonal) and r2(below the diagonal).**
(DOCX)
