# Supplementary material for: Investigation of Variants in UCP2 in Chinese Type 2 Diabetes and Diabetic Retinopathy
Source: PLoS One. 2014 Nov 14;9(11):e112670. doi: 10.1371/journal.pone.0112670 (PMC4232517; doi:10.1371/journal.pone.0112670)
Supplement: Table S1 — Primers used in PCR for each of the two polymorphisms. (DOCX) [file pone.0112670.s001.docx]

**Table S1. Primers used in PCR for each of the two polymorphisms.**

| **Polymorphisms** | **Primer** | **Sequence (5'-3')** |
| --- | --- | --- |
| rs660339 | Upper | GCTCAGAGCCCTTGGTGTAG |
|  | Lower | CCTGGGAGTCTTGATGGTGT |
| rs659366 | Upper | TGACCTCACGCTCCTACACA |
|  | Lower | ACTTCTGTTTCCACGCTGCT |
