# Supplementary material for: Investigation of Variants in UCP2 in Chinese Type 2 Diabetes and Diabetic Retinopathy
Source: PLoS One. 2014 Nov 14;9(11):e112670. doi: 10.1371/journal.pone.0112670 (PMC4232517; doi:10.1371/journal.pone.0112670)
Supplement: Table S2 — Hardy-Weinberg equilibrium testing. (DOCX) [file pone.0112670.s002.docx]

**Table S2. Hardy-Weinberg equilibrium testing.**

| **Polymorphism** | **Controls** | **DM** | **DWR** | **DR** | **NPDR** | **PDR** |
| --- | --- | --- | --- | --- | --- | --- |
| rs660339 | χ2=2.46 | χ2=0.97 | χ2=2.57 | χ2=0.01 | χ2=0.50 | χ2=0.22 |
|  | P=0.12 | P=0.33 | P=0.11 | P=0.93 | P=0.48 | P=0.64 |
| rs659366 | χ2=1.97 | χ2=0.57 | χ2=0.06 | χ2=0.41 | χ2=0.36 | χ2=1.16 |
|  | P=0.16 | P=0.45 | P=0.81 | P=0.52 | P=0.55 | P=0.28 |
