# Supplementary material for: Investigation of Variants in UCP2 in Chinese Type 2 Diabetes and Diabetic Retinopathy
Source: PLoS One. 2014 Nov 14;9(11):e112670. doi: 10.1371/journal.pone.0112670 (PMC4232517; doi:10.1371/journal.pone.0112670)
Supplement: Table S3 — Multiple comparison of genotypes distribution in rs660339 between DM and control. (DOCX) [file pone.0112670.s003.docx]

**Table S3. Multiple comparison of genotypes distribution in rs660339**

**between DM and control.**

|  | **DM** | **Control** | **χ2** | **P** |
| --- | --- | --- | --- | --- |
| CC | 166 | 121 | 10.688 | 0.001 |
| CT | 87 | 116 |  |  |
|  |  |  |  |  |
| CT | 219 | 204 | 4.364 | 0.037 |
| TT | 87 | 116 |  |  |
|  |  |  |  |  |
| CC | 166 | 121 | 2.535 | 0.111 |
| CT | 219 | 204 |  |  |
