# Supplementary material for: Investigation of Variants in UCP2 in Chinese Type 2 Diabetes and Diabetic Retinopathy
Source: PLoS One. 2014 Nov 14;9(11):e112670. doi: 10.1371/journal.pone.0112670 (PMC4232517; doi:10.1371/journal.pone.0112670)
Supplement: Table S4 — Linkage disequilibrium between SNPs in our samples, showing D'(above the diagonal) and r2(below the diagonal). (DOCX) [file pone.0112670.s004.docx]

**Table S4. Linkage disequilibrium between SNPs in our samples,**

**showing D'(above the diagonal) and r^2^(below the diagonal).**

|  | **rs660339** | **rs659366** |
| --- | --- | --- |
| **rs660339** | — | 0.519 |
| **rs659366** | 0.254 | — |
